# Sex Differences in the Neurobiology of Alcohol Use Disorder

**DOI:** 10.35946/arcr.v40.2.04

**Published:** 2020-10-08

**Authors:** Annabelle Flores-Bonilla, Heather N. Richardson

**Affiliations:** 1 Neuroscience and Behavior Program, University of Massachusetts, Amherst, Massachusetts; 2 Department of Psychological and Brain Sciences at the University of Massachusetts, Amherst, Massachusetts

**Keywords:** alcohol use disorder, animal models, sex differences, stress, adolescence, alcohol, brain

## Abstract

Sex differences may play a critical role in modulating how chronic or heavy alcohol use impacts the brain to cause the development of alcohol use disorder (AUD). AUD is a multifaceted and complex disorder driven by changes in key neurobiological structures that regulate executive function, memory, and stress. A three-stage framework of addiction (binge/intoxication; withdrawal/negative affect; preoccupation/anticipation) has been useful for conceptualizing the complexities of AUD and other addictions. Initially, alcohol drinking causes short-term effects that involve signaling mediated by several neurotransmitter systems such as dopamine, corticotropin releasing factor, and glutamate. With continued intoxication, alcohol leads to dysfunctional behaviors that are thought to be due in part to alterations of these and other neurotransmitter systems, along with alterations in neural pathways connecting prefrontal and limbic structures. Using the three-stage framework, this review highlights examples of research examining sex differences in drinking and differential modulation of neural systems contributing to the development of AUD. New insights addressing the role of sex differences in AUD are advancing the field forward by uncovering the complex interactions that mediate vulnerability.

## BACKGROUND

Addiction is a chronic relapsing disorder characterized by continued substance misuse despite harmful consequences. Alcohol use disorder (AUD) is specific to the maladaptive consumption of alcohol.^1^,^2^ The fifth edition of the *Diagnostic* *and Statistical Manual of Mental Disorders* (DSM-5), published by the American Psychiatric Association, describes AUD by mild, moderate, and severe subclassifications depending on the number of criteria met for the diagnosis.^3^ These criteria include symptoms of (1) compulsive excessive drinking; (2) persistent desire to consume alcohol and unsuccessful efforts to quit; (3) increased time spent in activities necessary to obtain, consume, and recover from alcohol; (4) craving or strong desire to consume alcohol; (5) recurrent use of alcohol that disrupts obligations such as work, school, or home; (6) continued use of alcohol despite persistent social or interpersonal problems; (7) important social, recreational, or occupational activities are reduced; (8) drinking persists in situations that cause harm to the individual or others; (9) consumption persists despite knowledge of the detrimental effects caused by alcohol; (10) tolerance for alcohol by having a diminished effect with the same amount or needing increased amounts for the same effect; and (11) symptoms of alcohol withdrawal. Mild AUD meets two or three of the criteria, moderate AUD meets four or five of the criteria, and severe AUD meets six or more of the 11 total criteria. The severity diagnosis for AUD could be useful for determining distinct neurobiological profiles that may be associated with mild, moderate, and severe AUD. Importantly, preclinical and clinical studies that include sex as a biological factor in experimental design will be essential to fully understand these complex neurobiological mechanisms.

## OVERVIEW

The goal of this review is to discuss AUD using the three-stage framework of addiction—binge/intoxication, withdrawal/negative affect, and preoccupation/anticipation^4^—to highlight examples of sex differences in drinking and related behaviors and to describe some of the neurobiological systems underlying AUD. There has been a recent upsurge in clinical studies in humans and experimental studies in animals in which females are included in the experimental design to elucidate the role of sex in the transition from alcohol use, to alcohol misuse, and ultimately to AUD. Sex differences may influence the three phases of addiction and consequently impact AUD risk differently in men and women.^5^ The approach of considering sex as a biological factor in study design has gained even more traction because the gap between men and women in the prevalence of AUD has been closing in the past few years.^6^,^7^

This review focuses primarily on preclinical animal studies using self-administration procedures to elicit alcohol exposure and/or to measure drinking behaviors to allow for more direct comparison to key findings about drinking behaviors in humans. Preclinical drinking models are summarized in other reviews.^8^–^12^ This article also considers the implications of sex on the onset of drinking, the exacerbation of the negative consequences of drinking, and the increased cue-induced relapse in more advanced stages of AUD. Overall, by presenting examples of studies that address sex differences within these stages, this review aims to show the dynamic role sex differences may have on vulnerability to the development of AUD, to generate enthusiasm for studying sex differences in preclinical and clinical alcohol research, and to advance our understanding and treatment of AUD.

## BINGE/INTOXICATION STAGE

In this phase, individuals consume enough alcohol to induce intoxication and cause impairment of physical and mental abilities. An example of this is binge drinking—the excessive consumption of alcohol that results in blood alcohol levels of 0.08 gram percent (g/dL) or higher—typically reached by consumption of five or more drinks in men and four or more drinks in women within a 2-hour period.^12^–^15^ When individuals first start binge drinking, they may not experience any physiological or emotional changes of withdrawal when the alcohol wears off; however, this changes over time.

### AUD Prevalence and Age at Drinking Onset

The lifetime prevalence of AUD is 29% in the United States, with a higher prevalence in men than women.^2^ In the United States, 33% of men and 17% of women binge drink at least once a month, and longitudinal studies suggest that this gap is narrowing due to a decline in frequency among men.^15^ Sex differences in AUD prevalence may relate to the age at drinking onset or an individual’s first experiences with drinking alcohol—especially if alcohol consumption is high enough to elicit intoxication.^16^,^17^ The lifetime risk of AUD quadruples when drinking begins on or before age 14 versus age 18,^18^ and the factors motivating individuals to first start drinking and to drink heavily differ with sex.^16^,^17^

Higher risk-taking tendencies can lead to early-onset use and subsequent alcohol misuse—especially in males.^17^ Adolescent boys reported “risk taking” and “curiosity” as motivators for drinking alcohol, whereas this was not the case in adolescent girls.^17^ Adolescent boys also have higher levels of impulsivity and sensation seeking compared to adolescent girls.^19^ Likewise, men have lower aversion to risk in a social context compared to women, which may lead men to engage in more risk-taking behaviors.^20^ Interestingly, a significant positive relationship between sensation seeking and alcohol-related risks such as driving under the influence has been observed in women, but not men.^19^ This suggests that women with high sensation-seeking tendencies may have an increased chance of causing harm to themselves and others after drinking alcohol compared to men with the same sensation-seeking tendencies. Alcohol-induced increases in risk-taking behavior also have been shown to differ by sex in rodents, with adolescent male rats engaging in higher risk-taking behavior after drinking alcohol compared to adolescent female rats.^21^

Another reason that individuals may drink alcohol is for its acute anxiolytic, or anxiety-reducing, properties. Experimenter-administered alcohol intoxication can temporarily reduce anxiety-like behavior in rodents.^22^ Adolescent girls are more likely than adolescent boys to report drinking alcohol to alleviate stress, social isolation, and psychological distress.^23^ Similarly, female mice are more sensitive to the anxiolytic effects of experimenter-administered alcohol compared to males, indexed by increased time spent in the open arms of an elevated plus maze.^24^ Notably, the anxiety-reducing properties of alcohol are short-lived, experienced only during and immediately following alcohol drinking. As discussed later, and previously reviewed,^25^ there is a rebounding effect during the withdrawal phase after alcohol wears off, and the degree of negative affect and altered stress hormone levels experienced at that time differs with sex.

Overall, these studies suggest that sex plays a distinct role in the motivating factors leading to drinking initiation. Risk-taking behaviors are more likely to influence adolescent boys to consume alcohol, whereas adolescent girls are more likely to consume alcohol due to its anxiety-reducing properties. Understanding the factors underlying early alcohol drinking onset may produce better strategies to prevent and dissuade alcohol consumption in adolescence and may help create specialized alternatives to alleviate the need for this coping mechanism.

### Frontal Lobe Development and Early-Onset Drinking

Drinking during adolescence has been shown to lead to higher levels of drinking in adulthood in both male and female mice.^26^ Heightened levels of risky behavior, such as binge drinking, during adolescence is thought to occur, at least in part, because the frontal lobes are still undergoing significant development during this time. Through its connections to other cortical regions and subcortical limbic structures, the prefrontal cortex coordinates higher executive function and behavior including decision making, stress responses, working memory, and attention.^9^,^27^–^29^ The anterior cingulate cortex is one of the medial prefrontal regions that is negatively impacted by alcohol drinking, with more pronounced effects in adolescent male rodents and young men compared to adolescent female rodents and young women.^30^–^32^

Imaging studies in humans show other prefrontal regions are also altered with alcohol drinking in adolescence and early adulthood. The dorsolateral prefrontal cortex is thinner in younger adults who frequently engage in heavy drinking (≥5 drinks) compared to controls, and the magnitude of this effect is more robust in young adult men compared to young adult women.^32^ Binge drinking is associated with lower cortical volume and thickness in adolescent boys versus higher cortical volume and thickness in adolescent girls.^33^–^35^ Notably, alcohol-naïve adolescent boys and girls with a family history of AUD have thinner orbitofrontal cortices compared to age-matched adolescents without a family history of AUD, indicating that some cortical differences precede alcohol misuse.^36^ Considering these findings altogether, it is conceivable that an underdeveloped prefrontal cortex may promote early-onset of alcohol drinking, which could further delay or perturb this development—especially in boys and young men—and increase their lifetime risk of developing AUD.

### Gonadal Hormones and Dopamine

Reward comprises learning (cue associations), hedonic (“liking”), and motivational (“wanting”) components.^37^ Conditioned stimuli are initially associated with a reward, but can become motivational cues on their own, incentivizing both appetitive approach and consummatory behavior.^37^,^38^ Female rats show more appetitive approach, measured by the total number of head entries into a dipper access area (dipper approaches) and have higher levels of lever presses (active lever approaches) to obtain the alcohol reward.^39^ Consummatory behavior, measured by the number of dipper presentations into the access area (reinforcers delivered) is also higher in female rats compared to male rats.^39^ This is consistent with other rodent studies showing that females consume more alcohol relative to body weight and engage in higher levels of cue-mediated alcohol-seeking behaviors compared to males.^40^–^42^

The mesocorticolimbic dopamine pathway may contribute to sex differences in appetitive and consummatory behaviors, given its essential role in conditioning and associative learning of environmental and physiological cues that predict alcohol reward availability.^39^,^43^–^45^ Alcohol binge drinking activates cells in the ventral tegmental area (VTA) of the mesocorticolimbic dopamine pathway.^45^–^47^ This midbrain structure is the origin of dopaminergic cells that project to the ventral striatum (nucleus accumbens), frontal cortex, and amygdala. Rats will press a lever to self-administer alcohol directly into the VTA, but a higher dose of alcohol is needed for reinforcement of this behavior in males compared to females.^48^,^49^ Moreover, a prior history of adolescent intermittent alcohol exposure leads to heightened sensitivity to the rewarding properties of alcohol in both sexes, indexed by a leftward shift in alcohol dose-response curves in rats.^48^ In humans, a familial history of AUD is associated with an exaggerated ventral striatum dopamine response to the expectation of alcohol.^50^ Although this study did not find a sex difference in this dopamine response, perhaps a larger number of subjects would be needed to detect a subtle, but statistically significant, difference in this measure in men and women.^50^ Nevertheless, it is important to consider how dopamine contributes to sex differences in AUD vulnerability, given the role dopaminergic cells in the VTA play in reinforcement learning and in expectation of alcohol availability.

The interaction between gonadal hormones and dopamine may provide insight into the molecular mechanisms underlying sex differences in the rewarding properties of alcohol.^51^,^52^ Estradiol enhances the stimulating effect of alcohol on VTA dopamine neurons.^51^ In vitro extracellular recordings of dopaminergic neurons have been conducted using VTA slices obtained from female mice under the following hormonal conditions: no estradiol (ovariectomized and vehicle-treated) or low circulating levels of estradiol (gonadally intact mice in estrus) versus moderate (gonadally intact mice in diestrus II) or high (ovariectomized mice treated with proestrus-like levels of estradiol benzoate) circulating levels of estradiol.^51^ Alcohol increased excitation of VTA dopamine neurons in brain slices from mice of all hormonal conditions, but the effects were most robust when estradiol levels were moderate or high.

Lastly, in vitro treatment with ICI 182,780—an antagonist of estrogen receptor subtypes alpha and beta (ERα and ERβ, respectively)—attenuated alcohol-induced excitation of VTA dopamine neurons in mice with moderate levels of estradiol (diestrus II); this suggests that estradiol’s modulation of dopamine sensitivity to alcohol may be occurring through its acute interaction with ERα and/or ERβ subtype in the VTA slice. The acute interaction between estradiol and its receptors appears to depend on moderate or high estradiol levels, as the ERα/ERβ antagonist did not measurably attenuate alcohol-induced increases in dopamine firing under conditions of low estradiol (estrus).

Through its effects on mesocorticolimbic dopamine, estradiol appears to mediate association-based learning and the rewarding properties of alcohol in context, which could ultimately promote drinking. Indeed, estradiol-treated ovariectomized mice show both increased dopamine signaling in the VTA in response to alcohol and increased preference of an alcohol context compared to vehicle-treated ovariectomized mice.^53^ The preference for an alcohol-paired context suggests that estradiol enhances the rewarding effects of alcohol.^53^ Estradiol also increases alcohol consumption in these mice and inhibition of either ERα or ERβ blocks this effect, suggesting that co-activation of both receptor subtypes is dependent on estradiol.^53^

Progesterone and its metabolites also have been implicated in the modulation of mesocorticolimbic dopamine neurons in response to alcohol.^54^ A study in male rats showed that progesterone increases the dopamine extracellular concentration in the medial prefrontal cortex after an experimenter delivered administration of alcohol, inducing a 55% increase compared to controls.^54^ Alcohol intake also increases brain concentrations of allopregnanolone (3a-hydroxy-5a-pregnan-20-one)—a neuroactive metabolite of progesterone.^55^ Nonhuman primate research in females shows that drinking levels increase when serum levels of estradiol and progesterone and its metabolites are higher (i.e., during the luteal phase compared to the follicular phase of the menstrual cycle).^56^ Within the luteal phase the highest drinking occurred on the declining phase of the progesterone peak, with a trend of a positive correlation between serum allopregnanolone levels and alcohol intake.^56^ Progesterone and neuroactive steroids could be modifying drinking behavior through effects on mesocorticolimbic dopaminergic neurons involved in reward processing, but more research is needed to understand sex differences in these effects.^54^

### Sensitivity to the Aversive Consequences of Drinking

Binge drinking can cause injuries and other adverse outcomes, with high-intensity (extreme binge) drinking (10 or more drinks in men, eight or more drinks in women) resulting in more severe consequences such as blackouts, alcohol overdose, and even death.^57^ Some of the short-term aversive consequences of alcohol intoxication can help curtail continued alcohol consumption; yet, these are more subdued during adolescence, and in males in particular.^57^ Adolescent boys are less prone to the negative effects of alcohol after a binge-drinking episode, taking less time to recover from alcohol intoxication compared to adolescent girls.^23^ Similar trends of decreased sensitivity to the aversive properties of alcohol have been reported in male rodents, but this varies with age, species, and other factors.^58^–^61^ Nevertheless, reduced sensitivity to the aversive properties of alcohol may contribute to higher levels of binge and extreme binge drinking in adolescent boys compared to adolescent girls, which ultimately could lead to differential risk of AUD in adulthood.^57^

## WITHDRAWAL/NEGATIVE AFFECT STAGE

After repeated episodes of binge drinking, individuals can begin to experience a negative affective state when alcohol is withdrawn voluntarily or involuntarily. This includes dysregulated stress hormone levels, dysphoria, anxiety, depression, and irritability—a symptomology thought to be due in part to adaptations in stress-related neural pathways.^9^,^62^,^63^ Experiencing these aversive symptoms when alcohol wears off can set up a strong cyclical pattern of negative reinforcement in which individuals learn that if they consume alcohol again, they can “feel normal”—at least temporarily.

### Negative Affective State During Alcohol Withdrawal

Chronic heavy alcohol consumption eventually can lead to severe AUD. A hallmark feature of AUD is the negative emotional and physiological state that arises when alcohol wears off.^64^ Individuals may experience a combination of various symptoms ranging from dizziness to headaches, irritability, anxiety, dysphoria, sleep disturbances, and hypersensitivity to pain.^3^ As mentioned above, it has been proposed that alcohol dependence arises because individuals go through repeated cycles in which alcohol consumption serves to mediate the effects of withdrawal, acting as a negative reinforcer.^5^,^25^,^45^,^65^,^66^ A negative reinforcer is a driving force that—with the removal of an aversive stimulus such as negative affective state during withdrawal—promotes a specific behavioral response such as drinking relapse.^65^

Individuals with AUD report having negative and unpleasant feelings during withdrawal, such as low self-concept, neuroticism, depression, and hostility—all of which predict alcohol craving.^67^,^68^ Behavioral assays also have been developed to assess a negative affective state experienced during withdrawal in animals. In addition to the traditional assays such as the elevated plus maze and open field, the frequency of ultrasonic vocalizations also can be measured to assess anxiety-like symptoms of negative affect that are experienced early after withdrawal from chronic alcohol exposure in rodents.^69^,^70^ A recent study used this measure to examine sex differences in withdrawal-induced negative affect in rats that were exposed to 6 weeks of intermittent alcohol.^71^ The researchers found that male rats increased the frequency of vocalizations during acute withdrawal, whereas female rats did not.^71^ A difference in withdrawal sensitivity may incentivize continued heavy alcohol use to a greater degree in males compared to females, thus putting them at a higher risk of AUD.

Male rats and mice show a more pronounced display of negative affective-like behaviors and neuroactivity after withdrawal from chronic alcohol exposure compared to female rats and mice.^71^–^75^ Alterations in glutamate signaling from the stria terminalis projecting into the basolateral amygdala are thought to mediate these behavioral differences.^73^,^76^ Shorter duration of exposure to chronic intermittent alcohol vapor intoxication and withdrawal cycles was sufficient to detect these synaptic alterations in male rats versus female rats.^73^ Furthermore, a translational study using magnetic resonance spectroscopy showed that rats exposed to chronic intermittent alcohol vapors and people diagnosed with AUD have increased glutamatergic neurotransmission during acute alcohol withdrawal compared to their respective controls.^77^

### Dysregulation of Stress Hormones

Withdrawal from alcohol is associated with a dysregulation of stress hormones. The hypothalamic pituitary adrenal (HPA) axis governs the neuroendocrine response to stress by releasing corticotropin-releasing factor (CRF) from the hypothalamus, which activates the release of the adrenocorticotropic hormone (ACTH) from the anterior pituitary, resulting in the release of the glucocorticoids from the adrenal glands (cortisol in primates and corticosterone in rodents).

Studies in humans show that, compared to men, women had lower ACTH and cortisol levels under baseline (resting) conditions in the morning, but were more sensitive to peripheral stimulation of the HPA axis as indexed by the dexamethasone/CRF test.^78^ In contrast, men showed a greater response than women to the centrally acting citalopram stimulation test.^78^ This test measures the extent to which a selective serotonin-reuptake inhibitor acts specifically on the hypothalamus to initiate a stress response. Compared to women, men also exhibited greater activation in response to stress of corticolimbic structures including the medial prefrontal cortex, the extended amygdala and posterior insula, and the hippocampus.^79^ In rodents, HPA activity is higher in females under basal (stress-free) conditions and in response to an acute stress challenge.^25^,^80^–^82^ In rodents, stress experienced in utero can exaggerate these sex differences even more by enhancing HPA responses in females and dampening it in males.^83^

In male rats, dampened HPA responsivity has been observed after withdrawal from chronic intermittent alcohol vapor exposure, and to a lesser extent following chronic alcohol drinking alone.^84^ Although sex differences in corticosterone responsivity were not directly tested, corticosterone responsivity appears to differ 24 hours into withdrawal from chronic alcohol drinking and following predator order stress in male and female mice.^81^ Studies in nonhuman primates and rodents have confirmed that alcohol drinking acutely elevates blood levels of ACTH and glucocorticoids.^81^,^84^–^86^ It is thought that repeated cycles of intoxication and withdrawal eventually desensitize this system, resulting in neuroendocrine tolerance to alcohol.^9^,^87^

Dysregulation of the HPA axis is thought to result from alcohol-induced neuroadaptive changes within this neuroendocrine axis itself.^84^ Glucocorticoid receptor signaling is required for the development of dependence, but it remains unknown whether the accompanying neuroendocrine tolerance contributes functionally to escalated drinking after dependence.^9^,^88^ In addition to the HPA axis, there are neuroadaptive changes in other stress regulatory pathways as well such as the prefrontal cortex, bed nucleus of the stria terminalis, and central amygdala.^9^,^47^,^88^–^91^

Stress can increase alcohol drinking, but this depends on sex, age, and the type of stress exposure.^81^,^92^ Adult female rodents show higher drinking compared to adult males, relative to body weight, and predator odor stress has been shown to elevate drinking in male rodents to the level of drinking observed in females.^40^,^80^ In one study, adult mice had 3 weeks of intermittent binge drinking using the scheduled high alcohol consumption (SHAC) procedure, followed by 1 month of abstinence, and then were tested for alcohol drinking before and following 2 weeks of intermittent predator odor stress (dirty bedding from rats).^81^ Among male mice with a prior history of binge drinking, 2 weeks of stress elicited the greatest increase in drinking relative to baseline. This stress effect was found in female mice only when the baseline drinking was stratified into two subgroups: low versus high levels of drinking. Only females that had originally exhibited low drinking levels showed the increase in drinking in response to stress.^81^ Female mice that initially exhibited high drinking did not show a further elevation, possibly due to a ceiling effect.

Another study of mice used the “Drinking in the Dark” (DID) binge drinking procedure for 2 weeks followed by 11 days of unpredictable, chronic, mild stress.^93^ Afterwards, alcohol drinking was measured with a two-bottle choice of 20% versus 40% v/v alcohol test. Stress increased alcohol binge drinking in both sexes, but this effect was exacerbated even more in male mice with a previous history of drinking prior to stress.^93^

The studies discussed above and others^94^ suggest that males may be more susceptible to alcohol withdrawal; however, early-onset drinking can interact with these factors and drive up vulnerability in females. Five days of exposure to restraint stress increased alcohol drinking in adolescent female rats, but decreased drinking in adolescent male and adult female rats.^92^ This suggests a heightened sensitivity to stress in adolescence that may have a particularly detrimental impact in females. In support of this, adolescent-onset binge drinking increased anxiety-like behavior early in withdrawal in female mice, and this persisted into abstinence.^95^ Likewise, acute stress elicited a negative affective state in the novelty-induced suppression of feeding task in adult female mice with a history of adolescent alcohol exposure.^76^ A history of adolescent binge drinking and intermittent alcohol vapor exposure led to a negative affective-like state in the elevated plus maze task and fear conditioning response in male mice, but it did not emerge until later in abstinence.^96^

The neural systems implicated in the interactive effects of stress and alcohol include not only structures of extended amygdala, but also brain regions thought to be involved in the third stage of AUD (preoccupation/anticipation).^73^,^86^,^97^–^100^ For example, a history of prior binge drinking and exposure to predator odor stress dysregulates protein levels of stress-related receptors, and does so in a sex-specific manner.^81^ After chronic drinking, there is a measurable increase in glucocorticoid receptors in the prefrontal cortex and hippocampus, and CRF receptor 1 in the hippocampus of female mice, but not male mice.^81^ These neuroadaptive changes in stress-regulatory circuits could persist well beyond withdrawal and underlie some of the psychological components that predict craving and relapse.^67^

## PREOCCUPATION/ANTICIPATION STAGE

Prolonged heavy alcohol use leads to a state of a constant preoccupation with alcohol and compulsive drinking despite negative consequences.^88^,^101^,^102^ This craving can continue into abstinence for months or years, making it difficult to abstain from alcohol altogether or to shift to a healthier level of drinking.^103^

### Sensitivity to Alcohol-Related Cues

After long bouts of abstinence, alcohol-related cues can trigger incentive salience, which heightens cravings and precipitates relapse.^37^,^104^,^105^ Men in particular exhibit higher levels of alcohol craving than do women,^106^ and cravings are associated with increased activity in the striatum in men, but not in women.^79^ Cue-induced reinstatement procedures are useful for studying the underlying neurobiological mechanisms by which alcohol-related cues promote craving and relapse during abstinence.^107^ Like humans, male rodents appear more susceptible to relapse than females.^108^ Brain-derived neurotrophic factor (BDNF) may play a role in mediating this sex difference.

In mice, male offspring of alcohol-exposed fathers have high *Bdnf* gene expression in the VTA and low alcohol drinking behavior; this effect was not observed in female offspring.^109^ Conversely, genetic manipulation to reduce BDNF protein levels to 50% in female rats resulted in a heightened, male-like, response to alcohol cues.^108^ This genetic manipulation had no effect in males. Others have found a sex difference in tropomyosin receptor kinase B (TrkB) signaling in *Bdnf* +/− mice, with males showing higher TrkB phosphorylation than females in the prefrontal cortex and striatum.^110^ Consequently, BDNF signaling is presumed to mediate cravings in response to alcohol cues and this increased sensitivity to alcohol-related cues could put males at higher risk of relapse even after long periods of abstinence.

### Compulsive Alcohol Drinking After Chronic Use

As discussed earlier, multiple cycles of binge intoxication followed by withdrawal can transition individuals from light to moderate drinking to severe AUD.^5^,^25^,^45^,^66^ At this point, heavy drinking can become more compulsive.^111^ Compulsive alcohol use is inflexible and persists despite negative consequences or despite devaluation of the rewarding effects of alcohol. This type of drinking is characteristic of physical and motivational/emotional dependence on alcohol.^88^,^112^

One strategy used to measure inflexible drinking is the assessment of a persistent motivation to drink despite increasing the response requirement to obtain alcohol. In animal studies, this can be tested by training subjects to press a lever or nose poke for alcohol in operant boxes.^9^ The number of responses to get the reward can be changed using fixed ratio or progressive ratio schedules of reinforcement in operant alcohol self-administration studies. Fixed ratio is the number of presses necessary for reward delivery, increasing the response requirement for the reward. This challenge measures compulsive-like behavior that is characteristic of addiction, in which individuals go to extreme lengths to obtain the drug on which they are dependent. Progressive ratio takes this a step further and increases the response requirement for reward delivery. In humans, a progressive ratio trial of intravenous alcohol self-administration showed that women increased their work effort to obtain alcohol after resumption following 2 weeks of abstinence, whereas men decreased this effort.^113^ Male rats exposed to alcohol vapors to produce dependence display increased compulsive-like behavior and increased intake on both fixed and progressive ratio schedules.^88^ However, progressive ratio tests in Long Evans rats suggest there is no sex difference in motivation for alcohol, at least following extinction and reinstatement of alcohol self-administration.^114^ Comprehensive studies are needed to assess compulsive drinking behaviors and relapse after prolonged abstinence in both nondependent and dependent animals to better understand sex differences in AUD.

Alcohol solutions also can be manipulated to devalue reward and to test for signs of inflexible drinking. One approach to devaluing alcohol is the addition of an unpleasant substance to change the flavor of alcohol by adding the bitter taste of quinine hydrochloride dihydrate or lithium chloride.^111^ Female mice have been shown to be more resistant to devaluation by quinine than males, and this sex difference was not attributable to differences in sensitivity to quinine.^115^ Nevertheless, sex differences in sensitivity to alcohol reward devaluation may be temperament- or species-specific, as male and female Long Evans rats reduce drinking levels to the same extent following alcohol devaluation.^114^,^116^ In addition to alcohol adulteration, more sophisticated procedures derived from behavioral economics can be used to manipulate the value of the reward by changing the alcohol reinforcer magnitude, availability of alternative reinforcers, and delay discounting.^117^,^118^

Another approach used to test for inflexible drinking is to measure shock-resistant alcohol intake.^112^,^119^ Rodent and human studies use these procedures to measure compulsive alcohol drinking despite negative consequences (e.g., foot shock or electric shock to the wrist, respectively). In rats, when one of eight alcohol-seeking responses are paired with foot shock, half of the alcohol-dependent male rats exhibit shock-resistant alcohol intake.^120^ Male alcohol-preferring rats that received an intermittent foot shock in response to alcohol seeking separated behaviorally into three distinct subgroups: (1) compulsive rats that continued alcohol seeking despite punishment, (2) noncompulsive rats that diminished their alcohol-seeking responses, and (3) an intermediate group that only partially suppressed their alcohol-seeking behavior.^119^ These two studies did not elucidate a sex difference as neither included female rats in the study design.^119^,^120^ Heavy alcohol use in men and women is associated with risky and inflexible drinking, with men and women with AUD making more attempts to obtain aversion-paired rewards compared to individuals without AUD.^121^,^122^ Furthermore, higher connectivity between the anterior insula and the nucleus accumbens is associated with increased compulsive-like behavior.^122^

Altogether, these studies suggest that inflexible drinking promotes heavy and continued use of alcohol and, consequently, may lead to further neuroadaptations in the brain. However, some of the devaluation strategies show limited evidence of sex differences. The inclusion of female subjects in these studies to directly compare the effects is vital to evaluate the role of sex in compulsive-like drinking under these different paradigms.

### Chronic Alcohol Use and Corticolimbic Circuitry

Deficits in executive function can result from early-onset drinking or chronic heavy use, and this may lead to a higher chance of relapse following abstinence.^123^ Some of these effects may be due to alterations in connectivity between prefrontal cortices and subcortical structures that are involved in reward processing.^5^,^124^ The medial prefrontal cortex, anterior insula, and striatum are more active and have stronger connections in men and women with AUD compared to controls.^125^ This could result in more subcortical control over decision-making processes based on reward reactivity rather than executive control.^125^

With long-term abstinence in both men and women, there is increased resting-state connectivity to brain regions that control executive function and decreased connectivity within reward processing regions.^126^ Connectivity between the nucleus accumbens and the orbitofrontal cortex has been observed to be stronger in individuals with a familial history of AUD compared to individuals without this predisposition.^127^ These studies suggest that chronic exposure to alcohol leads to reduced function of the prefrontal cortex, which, when combined with a stronger influence of striatal control over decision-making, can increase the risk of relapse.^125^,^127^

Animal studies have advanced our understanding of neural connectivity at the axonal and microstructural level, giving insight into the mechanisms by which prefrontal function improves across development and can be impaired after alcohol exposure. During adolescent development in rats, prefrontal axons undergo robust increases in myelin ensheathment, which corresponds with a twofold increase in neuronal transmission speed.^128^ Binge drinking during adolescence is also associated with altered neurodevelopmental trajectories including poor frontal white matter integrity in adolescent boys and girls.^129^,^130^

Longitudinal studies show that white matter growth is attenuated in the frontal lobes in humans who started drinking during adolescence—an effect that was comparable in both sexes.^131^,^132^ The abnormal microstructural development of white matter in the frontostriatal region relates to binge drinking during adolescence and poorer cognitive function.^133^,^134^ Likewise, animal studies show that voluntary alcohol exposure during adolescence decreases the density of myelinated axons in the anterior cingulate subregion of the medial prefrontal cortex, with higher adolescent drinking levels predicting lower working memory performance later in adulthood.^30^ Reduced myelin density was not observed in female rats after adolescent binge drinking,^31^ which corresponds with another study in mice showing that high doses of alcohol reduce myelin genes to a lesser extent in adolescent females compared to males.^135^

Despite more robust effects in males, examination of myelinated axons at the microstructural level shows that alcohol alters the nodal domain in both male and female rats.^31^ The nodes of Ranvier are the ion channel–rich gaps between myelin sheaths on the prefrontal axons, and reduced length-to-width nodal ratios were detected in male and female rats following adolescent binge drinking.^31^ In males, the decrease in nodal ratio was due to an increase in nodal diameter after the exposure, whereas in females it was due to a decrease in the nodal length. In both cases, these microstructural alterations have potential to negatively impact the speed and integrity of neural transmission, which is essential for effective communication within and between cortical and subcortical structures.^31^ Altogether these studies show alcohol affects cortical circuits that are important for executive functioning and behavioral control, and does so to a greater extent in males than in females.

Administration of extreme binge-like doses of alcohol damages the hippocampus and prefrontal cortex, and impairs memory in rats.^136^–^138^ While damage within the prefrontal cortex was similar in both sexes^138^ the severe damage to the dentate gyrus of the hippocampus was greater in females compared to males.^136^ The dentate gyrus is a subregion of the hippocampus where new granule neurons are normally produced for the formation of new memories; however, alcohol impairs cell proliferation and reduces the number of granule neurons in this region and does so to a greater extent in females.^136^ This damage is associated with a reduction of trophic support molecules and the heightened vulnerability in female rats appears to be due to more robust downregulation of BDNF, insulin-like growth factor 1 (IGF-1), and cyclic adenosine monophosphate (AMP) response element-binding protein (CREB) signaling cascades.^136^ These results are consistent with human studies in which the hippocampus was shown to be particularly vulnerable to the effects of alcohol binge drinking.^124^,^139^ Self-administration studies in rodents suggest that even much lower levels of alcohol (low-binge) can decrease neurogenesis and hippocampal size,^140^ with reports of alcohol drinking reducing neurogenesis to a greater extent in females compared to males^141^ or similarly in both sexes.^142^ Hippocampal damage after alcohol drinking in rodents corresponds with significant cognitive and memory dysfunction, especially when the alcohol exposure occurs during adolescence.^26^,^137^,^143^ Thus, early-onset drinking and chronic heavy alcohol use may eventually lead to sustained hippocampal damage to a greater extent in female rodents, which in conjunction with prefrontal dysfunction, could interfere with the ability to regulate reactivity to stress and alcohol-related cues that promote craving and relapse.

## CONCLUSIONS AND CLINICAL IMPLICATIONS

**Figure 1:**
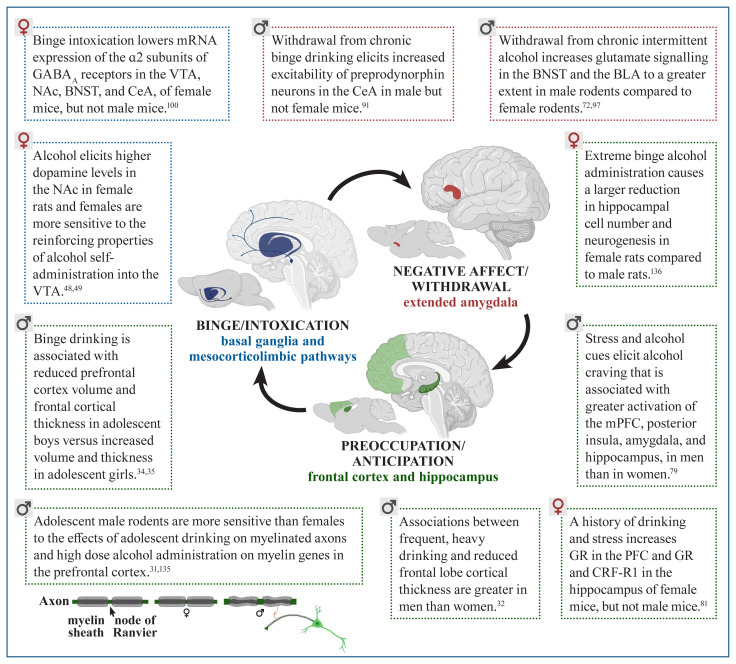
Sex differences in the effects of alcohol on the interacting brain systems associated with the three stages of addiction. *Note:* BLA, basolateral amygdala; BNST, bed nucleus of the stria terminalis; CeA, central amygdala; CRF-R1, corticotropin-releasing factor receptor 1; GABA_A_ receptors, gamma-aminobutyric acid type A receptors; GR, glucocorticoid receptors; mPFC, medial prefrontal cortex; mRNA, messenger RNA; NAc, nucleus accumbens; PFC, prefrontal cortex; VTA, ventral tegmental area. Created with BioRender.

The preclinical and clinical studies outlined in the current review show sex differences in behavioral risk factors and neural systems implicated in AUD, as summarized in Table 1 and Figure 1. This approach of incorporating sex differences in research studies has enhanced understanding of the complex mechanisms driving alcohol-related behaviors that lead to AUD. An increasing body of evidence shows sex differences in factors contributing to AUD vulnerability during the onset of alcohol drinking and later in the development of severe AUD and relapse following abstinence (see Table 1 for details).

Adolescent drinking in the context of stress, negative affect, and increased cue-reactivity is greater in females. Males show vulnerability with regard to higher levels of impulsivity and, compared to females, they are less sensitive to the aversive effects of intoxication, making males less likely to stop drinking. Sex also was found to be a predictor of the negative impact that chronic alcohol use has on the brain (see Figure 1 for details). Males show more severe reductions in cortical thickness and reduced myelinated fiber density in the prefrontal cortex, whereas females show more robust decreases in neurogenesis in the hippocampus in response to alcohol. Sex can specifically influence the effects of alcohol in the brain in the context of intoxication, withdrawal, and cravings, leading to a robust vulnerability to AUD. Overall, these findings show that sex differences in humans and animal models of AUD are also dependent on the unique physiological characteristics of the stages of addiction. Effects of alcohol can be mediated by sex in different directions, by increasing or decreasing vulnerability to AUD depending on the specific factor being considered. This complex shifting of vulnerability mediated by sex calls for a comprehensive approach toward studying AUD and other addictions.

A number of other health consequences endured after chronic heavy alcohol use are greater in women compared to men. Women with AUD experience higher risks of developing cancers, alcohol-related liver injury, and cardiovascular disease compared to men with AUD despite comparable levels of drinking.^7^,^25^,^144^–^150^ Specifically, binge drinking shows an increase of mortality, including cancer-related mortality, and people with AUD have a threefold increase of death and a higher risk of digestive diseases, dementia, cancer, and liver disease. Women with AUD show higher risk of liver disease-related mortality, with 71% of mortality in women compared to 64% in men.^146^ Sex differences in the effects of alcohol drinking may be explained in part by the role of gonadal steroid hormones in modulating a variety of functions in the brain. These functions include regulation of hypothalamus-driven social behavior;^151^ cognition, memory, and learning driven by the hippocampus and the prefrontal cortex;^152^ amygdala-mediated stress responses;^25^,^153^ dopamine-mediated reward;^51^ and synaptic plasticity.^154^ Moreover, alcohol binge drinking in women can dysregulate the menstrual cycle,^155^ which can affect endogenous steroid hormone levels.^156^–^159^

New diagnostic neuroimaging approaches are being explored to improve the assessment of AUD severity and circumvent limitations of the more traditional methods such as the Alcohol Use Disorders Identification Test (AUDIT) self-report questionnaire. A metabiological study recently reported that resting state connectivity functional magnetic imaging can be useful for assessing AUD.^160^ Specifically, differential functional connectivity between the prefrontal cortex and the reward-related areas predicted the severity of AUD with accuracy that surpassed other functional magnetic resonance imaging, structural magnetic resonance imaging, combined magnetic resonance imaging features, or demographic features. The usefulness of these new diagnostic approaches exemplifies the great urgency for more inclusion of female subjects in preclinical AUD studies in humans and animal models. With heightened attention to detail in experimental design and increased consideration of sex/gender differences in interpretation of research findings, we can enhance our understanding of the neurobiological mechanisms underlying AUD to improve diagnosis and treatment in the future.

## Figures and Tables

**Table 1:** Sex Differences in Behaviors Associated With the Three Stages of Addiction

Binge/Intoxication	
**Risk factors that promote early-onset drinking**	Impulsivity, a risk factor for adolescent drinking, is higher in adolescent boys compared to girls.^19^
	Drinking to alleviate psychological distress is higher in adolescent girls compared to boys.^23^
**Alcohol drinking behavior**	Prevalence of binge drinking is higher in adolescent boys compared to girls.^15^
	Appetitive approach in response to a dipper presentation is greater in female rats than male rats.^39^
	Acute alcohol injection increases preference to a large/uncertain reward (a measure of risk-taking behavior) in males, with no preference shown in females.^21^
**Withdrawal/Negative Affect**	
**Alcohol drinking behavior**	Restraint stress increases drinking in adolescent female rats, but decreases drinking in adolescent male rats.^92^
	A prior history of adolescent binge drinking augments drinking levels later in adulthood in female mice, but not in male mice.^81^
	Female mice drink more alcohol under baseline conditions in adulthood, but a history of binge drinking and chronic unpredictable stress or predator odor can elevate drinking in male mice to the level of females.^81^
**Effects of alcohol withdrawal on negative affect**	Adolescent girls report more negative mood states following recent heavy episodic drinking than do adolescent boys.^23^
	A history of adolescent binge drinking elicits active coping responses to stress in female mice vs. passive coping responses to stress in male mice (indexed by less time vs. more time immobile in the forced swim test).^95^,^96^
	Frequency of ultrasonic vocalizations, a measure of anxiety-like behavior, is increased following withdrawal from chronic intermittent alcohol vapors in male rats, but not females.^69^–^71^
**Preoccupation/Anticipation**	
**Alcohol drinking behavior**	Men exhibit higher levels of alcohol craving in response to cues than women do.^106^
	Women increased work effort in a progressive ratio trial following resumption after 2 weeks of abstinence. Men showed a decrease in effort.^113^
	Relapse-like behavior in response to alcohol availability is higher in male rats compared to female rats.^108^
	Female mice have a higher degree of aversion-resistant drinking than male mice.^115^
